# Intimate partner violence against HIV-Positive women on ART follow-up and associated factors in public health facilities of western Ethiopia

**DOI:** 10.1186/s12981-023-00542-y

**Published:** 2023-07-25

**Authors:** Shibiru Biranu, Motuma Getachew, Gemechu Kejela, Chaltu Kifilu

**Affiliations:** 1 Department of Public Health, Institute of Health Sciences, Wollega University, Nekemte, Ethiopia; 2 Department of Nursing and Midwifery, Institute of Health Sciences, Wollega University, Nekemte, Ethiopia

**Keywords:** Intimate partner violence, HIV/AIDS, Women, Ethiopia

## Abstract

**Introduction:**

Intimate partner violence is the most pervasive but less recognized problem which affects millions of women world. It is more common among marginalized individuals including women affected by HIV. However, there is limited information regarding this problem among HIV-Positive Women in Ethiopia. Thus, the study was aimed to assess the magnitude and factors associated with intimate partner violence among HIV positive women in western Ethiopia.

**Methods:**

A facility-based cross-sectional study was conducted among HIV-positive women on ART follow-up in Nekemte town. A total of 420 women were selected by the simple random sampling technique and an interviewer-administered questionnaire was used for data collection. The data were entered to EpiData version 3.1 and analyzed by SPSS version 20. Univariable and mult- variable logistic regression analysis with their corresponding odds ratio (95%CI) were computed, and statistical significance was declared at p < 0.05.

**Results:**

The magnitude of intimate partner violence among HIV-positive Women during Lifetime and since diagnosed with HIV was 49.29%, [(95% CI: 44.3–53.6%)] and 41.67%, [(95% CI: 37.1–45.7%)] respectively. Skipping daily ART medication ≥ 6 times/month [AOR = 3.56; (95% CI 1.18, 10.74)], experiencing controlling behavior by a partner[AOR = 6.37; (95% CI 3.26, 12.44)], women inter-parental witness of violence [AOR = 1.74; (95% CI 1.09, 2.79)], women having favorable attitude that justify wife-beating [AOR = 1.76; (95% CI 1.06, 2.94)]and non-disclosure of test result to partner [AOR 0.38; (95% CI 0.22, 0.66)] were factors associated with intimate partner violence since diagnosed with HIV.

**Conclusion:**

The magnitude of intimate partner violence among HIV-positive Women on ART follow-up was found to be high in the study area. Therefore, integrating intimate partner violence victim screening with ART services, empowering HIV-positive women, and increasing their awareness of sexual and reproductive rights is needed.

## Background

Violence Against Women(VAW) is a violation of fundamental human rights, which is rooted in gender inequality. It is a public health problem, and an impediment to sustainable development [1]. Intimate partner violence (IPV) is one of the most common forms of violence against women. It refers to behavior within an intimate relationship that causes physical, sexual, or psychological harm, including acts of physical aggression, sexual coercion, and psychological abuse, and controlling behavior [2]. IPV is now widely recognized as a serious human rights abuse [3]. While some women, including HIV positive women are more at risk than others, it can happen to any woman, in any country regardless of culture, religion, or economic status [4].

World Health Organization (WHO) has estimated that 30% of ever-partnered women globally have experienced physical and/or sexual IPV, calling IPV a public health problem of epidemic proportions [5]. Women experiencing Violence face a 50% greater risk of HIV infection. The intersection between the two creates a dual epidemic [6, 7]. Also, IPV is one of the barriers to Anti-Retroviral Therapy (ART) treatment initiation and adherence among HIV-positive women, which is a profound global challenge for health, development, and women’s rights [4].

Studies conducted in different part of the African countries imply that IPV among HIV positive women is a common phenomenon; The study conducted among HIV positive women in Uganda shows that, 32.1% of women experienced physical violence, 28.3% experienced sexual violence, and 44.2% experienced any IPV [8]. Also, a study conducted in Cameroon shows that, the prevalence of emotional violence was 29% while physical and sexual violence were 22% and 18% respectively [9]. A study conducted in Tanzania shows that 69% of respondents experienced emotional or physical violence, 31% experienced sexual violence and 65% reported a lifetime physical, emotional, or sexual violence [10]. In Ethiopia, a study conducted on HIV positive women on ART in Fitche, indicates that, the prevalence of lifetime IPV was 46%, while Physical, psychological, and sexual violence were 43.7%, 43.7%, and 25.1% respectively [11].

There are different predictors of experiencing IPV among HIV-positive women, including; partner age, presence of opportunistic infection, women’s history of alcohol use, partner’s history of a fight with another person, non-partner beating/physical mistreatment, controlling behaviors, alcohol drinking, interparental witness of violence, discosure of test result, and low income of women [8–15].

Violence negatively affects women’s physical and mental health and well-being. It has social and economic consequences and costs for families, communities, and societies [1]. Millions of women, girls, children, and young people who are exposed to or experience violence suffer a range of short and long-term consequences. These include physical injuries, mental health problems, a higher risk of non-communicable diseases, sexual and reproductive health problems [16]. Violence or fear of violence has been implicated as a barrier to women seeking HIV testing and disclosure of HIV status [12]. If the relationship is characterized by abuse, or significant power disparities, the woman’s ability to use these services and HIV infection prevention methods will be compromised. This leads to significantly lower ART use, lower ART adherence, and lower odds of viral load suppression among women [13].

To prevent intimate partner violence and its effects, national and international efforts were tried. Among such efforts; empowering women, transforming cultural and social norms related to gender, integrating VAW and HIV services, promoting and implementing laws and policies related to VAW, gender equality and HIV were the major strategies set globally to respond and address VAW and HIV/AIDS jointly. Even though such efforts were made, still the problem continues to be a major challenge and a threat to women’s empowerment [2, 6].

Although IPV and HIV/AIDS are a pervasive problem among women, different studies conducted in different countries including Ethiopia are not well targeted to HIV-positive women. So the main aim of this study was to determine the magnitude of intimate partner violence among HIV-positive women on ART, and identify its associated factors at Nekemte town public health facilities, western Ethiopia. The findings of the study will be vital for policy makers, program managers, service providers, and researchers.

## Methods

### Study design, setting and period

Facility based cross sectional study was conducted among 420 HIV positive women on ART follow-up at Nekemte public health facilities from March 1 to April 3, 2020. Nekemte town is located at 331 Kilo Meters to the western direction from Ababa; Ethiopia. It is administratively subdivided into six administrative divisions and one rural kebele. In the year 2019, there are 26,538 households. The total population of the town is 132,711 out of these 65,028 males and 67,683 are females. There are 4,098 under one year, 20,930 under five years, 4, 420 pregnant women, and 23,731 non-pregnant women [17]. Regarding health facilities, there are 2 health centers, one specialized hospital, one referral hospital, two non-govenmrntal organizations clinics (Marie stopes international Ethioipia and Family Guidance Association Ethiopia). Besides these, there are also different private clinics in the town. Among those, all govermental health facilities provide ART services.

### Population and eligibility criteria

All HIV-positive women who were in intimate relations and were taking ART from Nekemte town public health facilities were the source population; and all HIV-positive women aged 15–49 years who were in intimate relations and were taking ART from Nekemte town public health facilities during the study period, and fulfill the inclusion criteria were the study population.

HIV-positive women who have been in an intimate relationship and who had made at least one visit to the ART clinic before the study were included in the study. Seriously ill patients and patients who were unable to give responses were excluded from the study.

### Sample size determination and sampling procedure

The sample size was estimated using a single population proportion formula; by considering assumptions of the proportion of lifetime intimate partner violence among HIV-positive women Proportion = 46% from the study conducted in the Oromia region, Fitche, central Ethiopia [11], a marginal error of 5%, a non-response rate of 10, and a 95% confidence interval. After including the above assumptions into the formula, the final sample size becomes 420 women on ART follow-up. Clients on ART follow-up are those who take antiretroviral drugs regularly for the rest of their lives, because, ART cannot cure HIV infection.

Nekemte town has four governmental health facilities (two hospitals and two health centers). All the four governmental health facilities were included into the study. Then, the total sample size was proportionally allocated to those health facilities according to the number of patients fulfilling the eligiblity criteria found on their records. After the total numbers of the study participants from each facility were determined; computer-generated simple random sampling technique was used to select the study participants. Eligible women who fulfill the inclusion criteria were assessed for lifetime experience of IPV and since diagnosed with HIV.

### Measurements

*Intimate partner violence* (IPV) is the self-reported experience of one or more acts of physical, sexual, and/or emotional violence by a current or former partner. *Physical violence* was measured when women reported at least one experience of being slapped or having something thrown at her that could hurt her, being pushed or shoved, being hit with a fist or something else that could hurt, being kicked, dragged, or beaten up, being choked or burnt on purpose, and/or being threatened with, or, having a gun, knife or other weapons against her by the current or former partner. *Psychological violence/emotional violence* occurred when the women reported at least one emotional violence item from the four questions listed in the WHO multi-country women and violence study questionnaire. It occurred when someone says or does something to make a person feel stupid or worthless. *Sexual violence occured* when women reported at least one experience of the three sexual violence questions. “Being physically forced to have sexual intercourse against her will, having sexual intercourse because she is afraid of what her partner might do, and being forced to do something sexual she found degrading or humiliating.

*Favorable attitude* is when women agreed with at least one item of the six Women’s Attitudes towards partner beating assessment questions set from WHO multi-country Women and violence study. In this study, *HIV- positive women on ART follow-up* is defined as HIV- positive women who had made at least one visit to the ART clinic before. *ART treatment adherence/medication adherence* was defined as; starting HIV treatment, keeping all medical appointments, and taking HIV medicines every day and exactly as prescribed. A woman is said to be adhered to ART treatment, if she take her medication every day, exactly as prescribed, and keep all medication appointments. Other wise, she was categorized as ‘not adhered’. In this study, *controlling behavior* occurred when an intimate partner/husbands of HIV-positive women/the respondent attempts to conform them/women to their own needs or desires through physical, sexual, and/or physicological harm. Exposure to care giver violence was occurred when HIV-postivive women are violated by their care gives atleast once during their life time and/or since diagnosed with HIV/AIDS. *Non dislosure of test results to partner* occurred when HIV-positive women/respondents refuse to tell their HIV status to their intimate sexual partner, due to fear of violence.

### Data collection tools and procedure

A structured questionnaire was adapted from the WHO multi-country study on women’s health and domestic violence against women, and modified based on the study variables and local context. It was first developed in English; then translated to Afan Oromo (regional language) and retranslated back to English by experts in both languages.

The questionnaire was composed of; socio-demographic characteristics of HIV-positive women on ART follow-up and their partners, magnitude and frequency of physical, emotional, and sexual violence, controlling behaviors, factors related to IPV, like clinical and behavioral factors of both women and partner. The questionnaire contains a total of 13 IPV-related questions (6 physical IPV-related questions, 4 emotional IPV-related questions, and 3 sexual IPV-related questions) to assess the experience of women to the three constructs of IPV (physical, emotional, and sexual). Also, there are controlling behaviours related questions to determine the exposure of IPV.

Data was collected by four female nurses and supervised by 2 MPH holder supervisors. Two days training was given for data collectors and supervisors. Before the actual data collection, a pretest of the questionnaire was conducted to check consistency, understandability, and clarity of the tool. In addition, completeness, accuracy, and consistency of the filled questionnaire was checked each night by supervisors.

### Data processing and analysis

After the completeness of the collected data was rechecked, the data was entered into EpiData version 3.1. and exported to SPSS version 20 for statistical anaylsis. The descriptive statistics were computed using the univariate analysis and the findings were presented in the form of narration, tables and charts. Binary logistic regression analysis was done to select the candidate variables for the final model (multivariable logistic regression). At binary logistic regression analysis, variables having p-value less than or equal to 0.25 were selected to be a candidate for the multivariable analysis. At multivariable logistic regression analysis, variables having p-value less than 0.05 were considered as the predictors of intimate partner violence among HIV-positive women. Model fitness was checked by the Hosmer-Lemeshow goodness of fit test (P = 0.74). Multicollinearity was ascertained using the VIF.

## Results

### Socio-demographic characteristics of HIV-positive women on ART

A total of 420 HIV-positive women aged 15–49 years on ART follow-up were participated in this study making a 100% response rate. The mean age of respondents was 33.16 years (SD ± 6.60). Nearly half, 208 (49.52%) of the respondents were in the age group of 25–34 years. The Women’s partners’ mean age was 41 years (SD ± 8.20). Half, (50.24%) of women’s partners were in the age range of 35–44 years. More than half, 247(58.81%) of respondents were protestant and407 (96.90%) of them were living in urban areas. Regarding their education, 161, (38.33%) of the respondents had attended primary education (grades 1–8), while 165(39.29%) of their partners had attended secondary education (grades 9–12).

On the other hand, 392(93.33%) of the respondents were currently married and nearly three quarter 296(70.48%) of the respondents had ≤ 4 family members. One hundred eighty (42.86%) of the respondent’s occupation was housewives, while 170(40.48%) of the partners were government/NGO employers, and one hundred fifty-two, (36.19%) of respondents had > = 3500 ETB average monthly income. Two hundred fifty-three (60.24%) of the respondents reported that a major household decision was made by both respondent and husband jointly (Table 1).

**Table 1:** Socio-demographic characteristics of HIV-positive women on ART follow-up and their partners in Nekemte town public health facilities, Western Ethiopia, from March-April, 2020

Characteristics N = 420	Frequency	Percent (%)
**Age**		
15–24	26	6.19
25–34	208	49.52
35–44	144	34.29
45–49	42	10
**Residence**		
Urban	407	96.90
Rural	13	3.10
**Marital Status**		
Married	392	93.33
Living in informal union/cohabitation	28	6.67
**Family size**		
≤ 4	296	70.48
> 4	124	29.52
**Religion**		
Orthodox	131	31.19
Muslim	42	10
Protestant	247	58.81
**Partner age**		
15–24	2	0.48
25–34	75	17.86
35–44	211	50.24
45+	132	31.43
**Educational status**		
can’t read and write	69	16.43
Grade 1–8	161	38.33
9–12	118	28.10
College and above	72	17.14
**Partner educational status**		
can’t read and write	33	7.86
Grade 1–8	115	27.38
Grade 9–12	165	39.29
College and above	107	25.48
**Occupation**		
Unemployed	180	42.86
Merchant	120	28.57 16.67
Gov’t/NGO/employer	70	
Student	38	9.05
Daily laborer	12	2.86
**Occupation of Husband**		
Farmer	21	5
Gov’t/NGO employer	170	40.48
Merchant	146	34.76
Daily laborer	77	18.33
Student	6	1.43
**Income**		
< 1500	129	30.71
1500–2499	79	18.81
2500–3499	60	14.3
>=3500	152	36.19
**Household major Decision-making power**		
Women	124	29.52
Husband	43	10.24
Women and husband jointly	253	60.24

### Behavioral characteristics of the study respondents

Four hundred ten (97.62%) of the respondents had only one male sexual partner and half, 212(50.48%) of them had used condoms consistently within the last twelve months. Among the study participants, the majority, 404(96.19%) them and their partners 265(63.10%) never drank alcohol. Nearly half, 212(50.71%) of the women and their partner 190(45.24%) did not witness their father beat their mother. Three hundred fifty-five (84.52%) of the partner did not ever involve in physical fighting with another person (Table 2).

**Table 2:** Behavioral characteristics of HIV- positive women on ART follow-up in Nekemte town public health facilities and their partners, Western Ethiopia, from March-April, 2020

Characteristics N = 420	Frequency	Percent (%)
**No of the sexual partner(s)**		
1	410	97.62
>=2	10	2.38
**Consistence use of a condom**		
Yes	212	50.48
No	208	49.52
**Women Drink Alcohol**		
Yes	16	3.81
No	404	96.19
**Husband/Partner’sDrink Alcohol**		
Yes	155	36.90
No	265	63.10
**Women inter-parental witness of violence**		
Yes	143	34.05
No	213	50.71
Don’t know	64	15.24
**Partner inter-parental witness of violence**		
Yes	83	19.76
No	190	45.24
parents did not live together/don’t know	147	35
**Partner ever involved in a physical fight with another man**		
Yes	65	15.48
No	355	84.52

### Clinical characteristics of HIV-positive women on ART follow-up

Nearly three fourth, 306(72.86%) of the respondents had the most recent CD4 lymphocyte count greater than500 cells/mm3; whereas only, 26(6.19%) had less than 200 cells/mm3. Among women taking ART medication, 359(85.48%) of them had suppressed viral load ( < = 1000 copies/ml) and most of them, 338(80.48%) had no opportunistic infection. More than half, 245(58.33%) of patients were categorized under stage I WHO HIV/AIDS clinical classification, and 48 (11.43%) of them have skipped their medication more than twice in the past one month. Three hundred ninety, (92.85%) of the study participants reported that they know their partner’s HIV status and of which around two-thirds 282(67.14%) of their current or most recent partner was HIV-positive.

In this study disclosing once, HIV status to partners was 369(87.86%) while 310 (73.81%) had disclosed their results to other than their partners and 117(27.86%) of them did not get any support after disclosing their result to a person other than their partner. Besides, 109(25.95%) of them entered to worsen relationships with their partner after disclosure of their result to their partners (Table 3).

**Table 3:** Clinical characteristics of HIV- positive women on ART follow-up in Nekemte town public health facilities, Western Ethiopia, from March-April, 2020

Characteristics N = 420	Frequency	Percent (%)
**Viral loud**		
<=1000 copies/ml	359	85.48
> 1000 copies/ml	61	14.52
**CD4 count**		
< 200cells/mm3	26	6.19
200-350cells/mm3	30	7.14
351-500cells/mm3	58	13.81
> 500cells/mm3	306	72.86
**Presence of OI**		
Yes	82	19.52
No	338	80.48
**HIV/AIDS clinical stage**		
I	245	58.33
II	100	23.81
III	62	14.76
IV	13	3.10
**Adherence to ART drug**		
Less than or equal to two times/month	372	88.57
From 3–5 times/month	25	5.95
More than or equal to 6 times/ month	23	5.48
**Partner HIV status**		
Positive	282	67.14
Negative	108	25.71
Un known	30	7.14
**Result disclosure to husband**		
Yes	369	87.86
No	51	12.14
**Relationship of the family after result disclosure**		
Better	107	25.48
Worse	109	25.95
no change	204	48.57
**Result Disclosure other than Partner**		
Yes	310	73.81
No	110	26.19
**Support received after disclosure other than the partner**		
Nothing	117	27.86
Advice	201	47.86
Money	102	24.29

### Women’s attitudes towards wife-beating among HIV-positive women

To understand the attitudes of women toward partner beating, they were asked to give an opinion on why a man could beat his partner. Those who have a favorable attitude toward wife-beating among the interviewed women account for137 (32.62%). However, the women who have a favorable attitude toward wife-beating regarding the refusal of sexual relations in circumstances such as *sickness*, when she *does not want*, etc. were349 (83.10%)(Table 4).

**Table 4:** Women’s Attitudes towards partner beating of HIV-positive women on ART follow-up in Nekemte town public health facilities, Western Ethiopia, from March-April, 2020

Characteristics = 420		Frequency	Percent (%)
**Attitude toward wife beating composite score**	Agree	137	32.62
	Disagree	283	67.38
Beating Wife is normal if she does not complete her household work to his satisfaction	Agree	53	12.62
	Disagree	367	87.38
Beating Wife is normal if She disobeys him	Agree	93	22.14
	Disagree	327	77.86
Beating Wife is normal if She refuses to have sexual relations with him	Agree	50	11.90
	Disagree	370	88.10
Beating Wife is normal if She asks him whether he has other girlfriends	Agree	35	8.33
	Disagree	385	91.67
Beating Wife is normal if He suspects that she is unfaithful	Agree	58	13.81
	Disagree	362	86.19
Beating Wife is normal if He finds out that she has been unfaithful	Agree	72	17.14
	Disagree	348	82.86
**Attitude to refuse to have sex in some situations composite score**	Agree	349	83.10
	Disagree	71	16.90
Women can refuse sex when She doesn’t want	Agree	242	57.62
	Disagree	178	42.38
Women can refuse sex when He is drunk	Agree	278	66.19
	Disagree	142	33.81
Women can refuse sex when She is sick	Agree	299	71.19
	Disagree	121	28.81
Women can refuse sex when He mistreats her	Agree	286	68.10
	Disagree	134	31.90
Women can refuse sex when She suspects he has extra-marital sexual relations with another woman	Agree	259	61.67
	Disagree	161	38.33
Women can refuse sex when She suspects her husband has an STI/HIV	Agree	139	33.10
	Disagree	281	66.90

### Forms of IPV

The study participants responded to the occurrence of different forms of IPV (psychological, physical, and sexual) in their lifetime, and since they were diagnosed with HIV.

#### Psychological violence

**Fig. 1:**
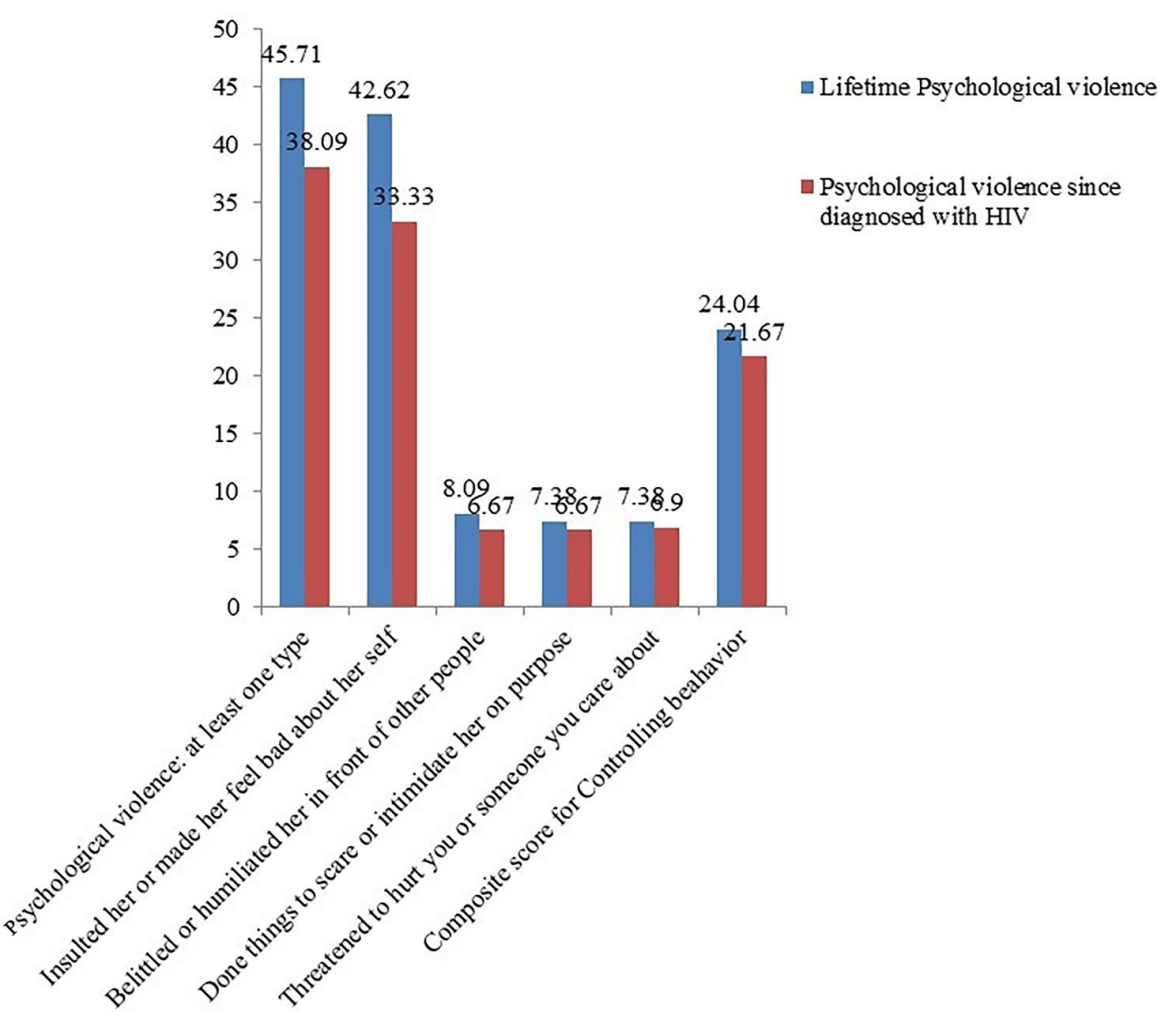
Magnitude of Psychological violence among HIV- positive women on ART follow-up in Nekemte town public health facilities, Western Ethiopia, from March-April, 2020

Among the study participants,179(42.62%) were *verbally insulted or made her feel bad about herself*, and 34(8.09%) were *belittled or humiliated by their partners in front of other people* in their lifetime, while 140(33.33%) and 28(6.67%) of the respondents experienced same abuse at least once since they diagnosed with HIV correspondingly. In addition to this, psychological violence was assessed separately for the existence of controlling behavior. Accordingly, either the *partner angry if she speaks with another man* and He is *often suspicious that she is unfaithful*, were 80(19.02%) and 71(16.90%) respectively in their lifetime and 73(17.38%) and 65(15.47%) respectively since diagnosed with HIV, were the most frequently reported controlling behavior from the listed items. The overall prevalence of controlling behavior among HIV-positive women on ART was 101(24.04%) for the lifetime and 91(21.67%) since diagnosed with HIV. Generally, the lifetime prevalence of psychological violence was 192(45.71%) and 160(38.09%) since diagnosed with HIV (Fig. 1).

#### Physical and sexual violence

Nearly one in five 87((20.71%) of women were slapped or thrown something at them and 33(7.86%) of women reported to have kicked, dragged, and beaten up in their lifetime, while 63(15%) and 25(5.95%) of same acts were reported since they diagnosed with HIV correspondingly. Nearly a quarter, 99(23.57%) of the women experienced at least one or more incidents of physical violence in their lifetime, and for 85(20.24%) study participants, the incidents have happened since they were diagnosed with HIV.

**Fig. 2:**
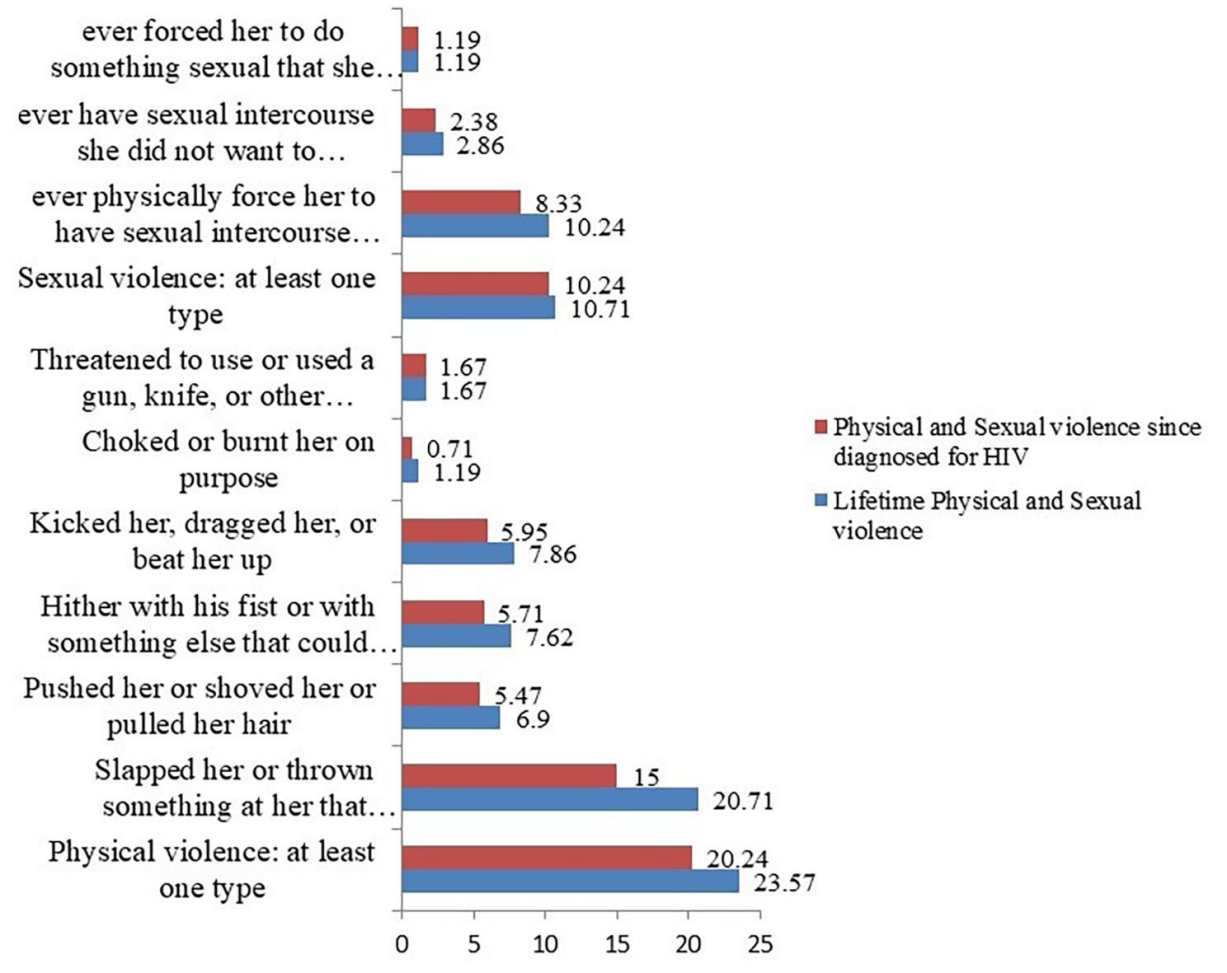
Magnitude of Physical and Sexual violence among HIV- positive women on ART follow-up in Nekemte town public health facilities, Western Ethiopia, from March-April, 2020

About 43 (10.24%) and 35(8.33%) of the respondents reported that their partners had forced them to have sexual intercourse without their interest in their lifetime and since they were diagnosed with HIV respectively. Besides, 12(2.86%) and 10(2.38%) respondents engaged in sexual intercourse in their lifetime and since they were diagnosed with HIV due to fear of their partners. Generally,45 (10.71%).and43(10.24%)of the respondents have reported having at least one incident of sexual violence in their lifetime and since diagnosed with HIV, respectively (Fig. 2).

### The magnitude of intimate partner violence

**Fig. 3:**
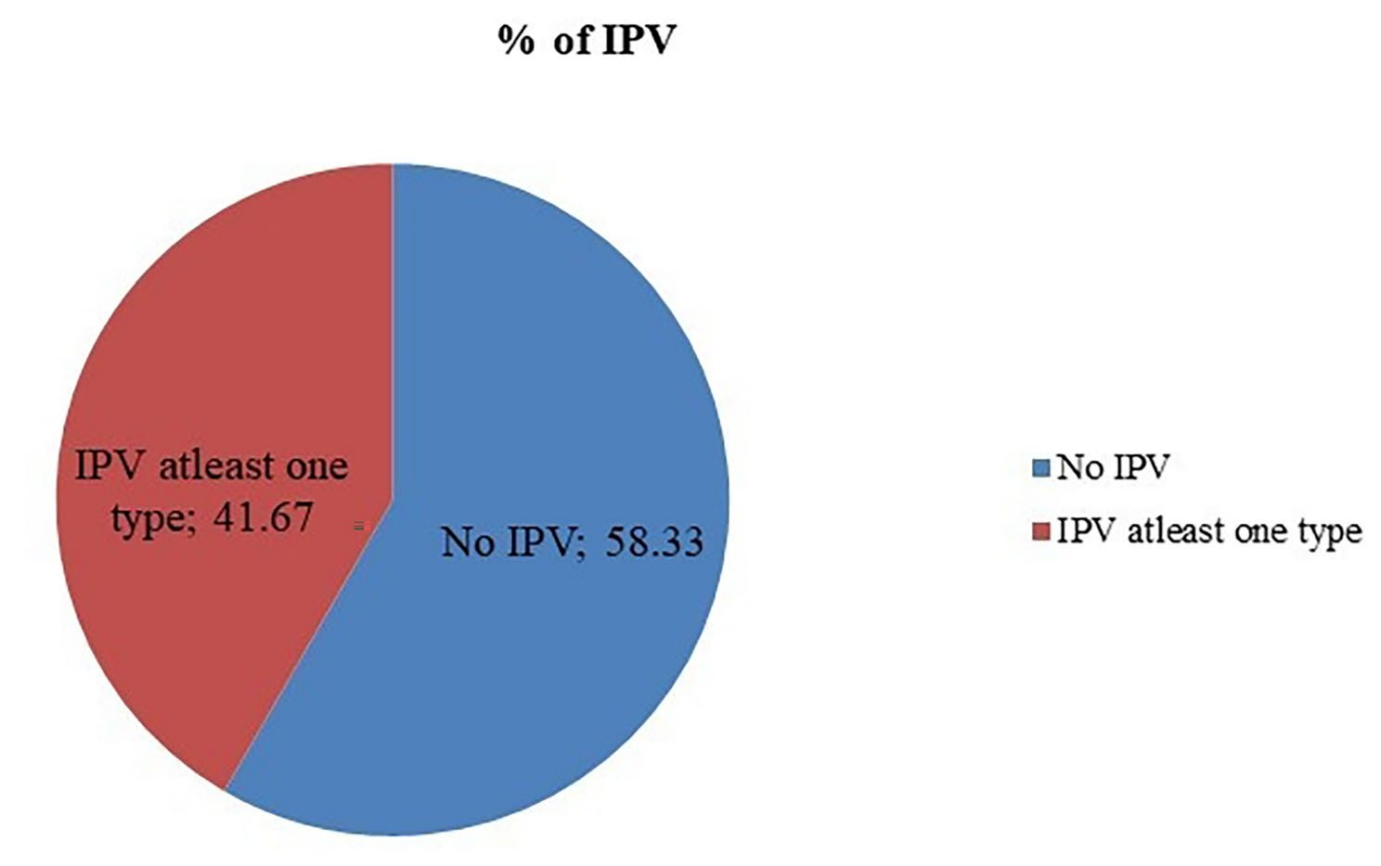
Magnitude of Intimate Partner Violence since diagnosed with HIV among HIV- positive women on ART follow-up in Nekemte town public health facilities, Western Ethiopia, from March-April, 2020

This study shows that nearly half, 207 (49.29%), [95% CI: 44.3–53.6%] of the respondents experienced at least one form of IPV (physical, sexual, Psychological) at some point in their lifetime and 175 (41.67) %, [95% CI: 37.4–45.7%] since diagnosed with HIV/AIDS respectively. Besides, the prevalence of lifetime physical injury by current or any other intimate partner was 9.5% (Fig. 3).

### Factors associated with intimate partner violence

To identify factors associated with intimate partner violence, the independent variables were analyzed in the bi-variable and multivariable logistic regression model. In binary logistic regression analysis, variables with a p-value of less than 0.25 were considered as a candidate variable and entered into the multivariable logistic regression analysis.

In multivariable logistic regression, variables like skipping ART medication ≥ 6 times, non-disclosure of HIV result to partners, controlling behavior, Inter-parental witness of violence, and having a favorable attitude toward wife beating had a p-value less than 0.05, and considered as a predictors of intimate partner violence among HIV-positive women on ART follow-up.

Women who are skipping their daily medication ≥ 6 times/month were 3.56 times [AOR = 3.56; 95% CI 1.18, 10.74] more likely to experience IPV compared to those who are skipping ≤ 2 times/month. Women who did not disclose their HIV results to partners were 62% [AOR 0.38; 95% CI 0.22, 0.66] less likely to experience IPV compared to their counterparts.

HIV-positive women who reported controlling behavior by an intimate partner were greater than six times [AOR = 6.37; 95% CI 3.26, 12.44] more likely to experience IPV compared to those who did not report controlling behavior. HIV-positive women who witnessed inter-parental violence were 1.74 times [AOR = 1.74; 95% CI 1.09, 2.79] more likely to experience IPV compared to their counterparts. HIV-positive women who have a favorable attitude that justifies wife-beating were nearly two and half times [AOR = 2.48 95% CI 1.51, 4.08] more likely to experience IPV compared to those who have an unfavorable attitude justify wife-beating in certain circumstances (Table 5).

**Table 5:** Factors associated with IPV among HIV-positive women on ART follow-up in Nekemte town public facilities, Western Ethiopia, from March-April, 2020

Characteristics (n = 420)	IPV after diagnosed With HIV		COR(95%CI)	AOR(95%CI)	
	Yes(%)	No(%)			
**Occupation of Husband**					
Governmental employer	27(39.13)	42(60.87)	1.00	1.00	
Farmer	45(36.89)	77(63.11)	0.91(0.50,1.67)	1.18(0.55,2.57)	
Merchant	62(42.47)	84(57.53)	1.15(0.64,2.06)	1.18(0.57,2.48)	
Daily laborer	41(49.40)	42(50.60)	1.52(0.80,2.90)	1.71(0.76,3.87)	
**Partner Drink Alcohol**					
Yes	81(52.26)	74(47.74)	2.00(1.33,2.98)	1.43(0.89,2.31)	
No	94(35.47)	171(64.53)	1.00	1.00	
**Condom use**					
Yes	76(35.85)	136(64.15)	1.00	1.00	
No	99(47.60)	109(52.40)	1.63(1.10,2.40)	1.38(0.85,2.23)	
**Women witnessed family violence**					
Yes	72(50.35)	71(49.65)	1.71(1.14,2.58)	1.74(1.09,2.79)*	
No	103(37.18)	174(62.82)	1.00	1.00	
**The husband/Partner witnessed family violence**					
Yes	49(59.04)	34(40.96)	2.21(1.28,3.83)	1.96(0.09,3.27)	
No	68(35.79)	122(64.21)	0.86(0.55,1.33)	1.11(0.65,1.88)	
parents did not live together	58(39.46)	89(60.54)	1.00	1.00	
**CD4 count**					
< 200 cells/mm3	16(61.54)	10(38.46)	2.38(1.05,5.42)	2.06(0.59,7.18)	
200–350 cells/mm3	12(40)	18(60)	0.99(0.46,2.13)	0.93(0.31,2.77)	
351–500 cells/mm3	24(41.38)	34(58.62)	1.05(0.59,1.86)	1.34(0.68,2.64)	
> 500 cells/mm3	123(40.20)	183(59.80)	1.00	1.00	
**WHO HIV/AIDS clinical stage**					
I	102(41.63)	143(58.37)	1.00	1.00	
II	36(36)	64(64)	0.79(0.49,1.28)	0.94(0.52,1.73)	
III	27(43.55)	35(56.45)	1.08(0.62,1.90)	1.01(0.46,2.23)	
IV	10(76.92)	3(23.08)	4.67(1.26,17.41)	2.46(0.42,14.46)	
**Adherence to ART drug**					
≤ 2 times/month	153(41.13)	219(58.87)	1.00	1.00	
From 3–5 times/month	6(24)	19(76)	0.45(0.18,1.16)	0.39(0.13,1.14)	
≥ 6 times/month	16(69.57)	7(30.43)	3.27(1.31,8.14)	3.56(1.18,10.74)*	
**Result Disclosure to Partner**					
Yes	174(47.15)	195(52.85)	1.00	1.00	
No	13(25.49)	38(74.51)	0.38(0.25,0.65)	0.38(0.22,0.66)**	
**Controlling behavior**					
No	111(33.74)	218(66.26)	1.00	1.00	
Yes	71(78.02)	20(21.98)	6.97(4.27,14.90)	6.37(3.26,12.44)**	
**Attitude toward wife beating**					
Unfavorable	103(36.40)	180(63.60)	1.00	1.00	
Favorable	72(52.55)	65(47.45)	1.94(1.28,2,93)	2.48(1.51,4.08)**	

Key: * = Statistically significant variables in multivariable logistic regression with P-value < 0.05

** = Statistically significant variables in multivariable logistic regression with P-value < 0.001

## Discussion

This study assessed the magnitude of IPV against HIV-Positive women on ART follow-up and associated factors in Nekemte town public health facilities, western Ethiopia. The lifetime magnitude of IPV was 49.29% [95% CI: 44.3–53.6%]. The most common forms of lifetime IPV were Psychological violence (45.71%); followed by physical (23.57%) and sexual violence (10.71%). This finding is nearly similar with the study conducted on HIV-positive pregnant women in Kinshasa, Democratic Republic of Congo reporting 51% IPV prevalence [18]. Also, the study conducted in Fitche hospital central Ethiopia reported 46% for lifetime IPV and 43.7% for psychological violence [11]. Additionally, the prevalence of a systematic review conducted in Ethiopia on the general population shows IPV prevalence ranged from 20 to 78% [19].

However this finding is lower than a study conducted at Columbia, Canada 60% for any IPV, 48% for physical, 55% psychological, and 27% for sexual violence [20]. A possible explanation for this variation may be a difference in the age group between the study populations that is, this study considers only women of reproductive age group (15–49), while the other study includes all women greater or equal to 19 years old. Also, the variation may be due to the difference between the sampling techniques of the study.

On the other hand, the magnitude of IPV since diagnosed with HIV/AIDS was 41.67% [95% CI: 37.4–45.7%]. This finding is nearly similar to the studies conducted in Wolayita zone, southern Ethiopia and Harare of Zimbabwe, which reported IPV among HIV-positive women of 44.2% and 40.5% respectively [8, 15].

Additionally, Psychological violence was the most common form of violence since diagnosed with HIV (38.09%), followed by physical violence (20.24%) and sexual violence (10.24%). It is in line with the study conducted in Wolaita, Ethiopia, which was 36.68% for psychological violence,28.57% for physical violence in the current/within past twelve months [21], and the magnitude of physical violence among HIV-Positive women in Cameroon was 22% [9]. In contrast, this finding is greater than the result from Osogbo, Southwest Nigeria conducted on HIV-positive women since HIV diagnoses; which was 23.6% for IPV, 17.2% for physical, 21.4% for psychological, and 2% for sexual violence [22]. This variation may be due to differences in the study setting and socio-economic differences among the study population.

The current study found that women witnessing inter-parental violence during their childhood increase the risk of experiencing IPV later in her life. This finding is in line with the research conducted in India and other countries [19, 23]. It has been suggested that witnessing inter-parental violence in early childhood has significant detrimental effects on the development of a child’s brain, which can lead to social, emotional, and behavioral problems. This might put them to be a victim by tolerating interpersonal violence in later life [16]. Also, it leads to a normative understanding of violence, and intergeneration learned behavior that is accepted as a means of conflict resolution [24].

Women who skip their daily medication ≥ 6 times/month were more likely to experience intimate partner violence. This result is in line with the previous research findings which indicate that IPV has a significant association with adherence to antiretroviral medication [13, 25]. This might be due to psychological disturbance that make a woman not adhere to her medication/not take her medication on time regularly.

Women who did not disclose their HIV status to their partners were less likely to experience violence. The finding of this result is also in agreement with the research conducted in Osogbo, southwest Nigeria, Tanzania, and other studies, which shows, HIV-status disclosure increases the risk of IPV in women living with HIV [15, 22, 26, 27]. It has been suggested that violence can be the consequence of Women living with HIV disclosing their HIV status and facing stigma and discrimination [6]. However, non-disclosure of the result cannot be the means to end violence, since it has grave public health implications, as it would promote the spread of the HIV/AIDS epidemic.

According to findings from this study, women who reported controlling behavior by intimate partners were at greater risk of experiencing IPV. This result is similar to other studies conducted in Fitche hospital, central Ethiopia, Wolaita Zone, Southern Ethiopia, a systemic review conducted in Ethiopia on the general population, and Harari Regional state, East Ethiopia [11, 19, 21, 28]. This might be a result of gender norms and roles, cultural practices, that collectively contribute to and perpetuate unequal power relations between women and men by encouraging male dominance and exercise control over their partner. When this occurs, it is difficult for women to negotiate within, or leave abusive relationships [6].

Women having a favorable attitude toward wife-beating or those who believe that partners should hit his wife in certain circumstances were more likely to experience violence. This is in line with other research findings conducted in Wolaita, Southern Ethiopia, Which reports Women who refuse sex in some situations were at a significant risk of being abused by their partner [21]. It may be due to the custom which can teach the women that violence is acceptable. Besides, women who believe that women deserve abuse in certain circumstances may be less likely to challenge male authority and therefore be learn to live with the abuse. However, the lack of sexual autonomy expressed by many women has substantial implications for women in the era of HIV/AIDS [6].

## Conclusions and recommendations

The findings of this study indicated that the magnitude of lifetime IPV and since diagnosed with HIV were found to be high compared to other previous studies. The most common form of IPV was psychological violence which was followed by physical and sexual violence respectively. The Coincidence of physical and psychological violence was the most reported feature among the overlaps of IPV.

Women’s inter-parental witness of violence, poor adherence to ART drug, non-disclosure of the result to Partner, controlling behavior, and favorable attitude toward wife-beating were factors associated with IPV. Therefore, integrating intimate partner violence victim screening with ART services, empowering HIV-positive women, and increasing their awareness of sexual and reproductive rights are needed. Furthermore, policy priority should involve intimate partner violence legal policy; involve males, and transform the culture of gender norms.

## Data Availability

The data sets used and analyzed for the current study are available from the corresponding author on reasonable request.

## Abbreviations

AIDS: Accrued Immune Deficiency Syndrome
ART: Anti Retroviral Therapy
CEDAW: Convention on the Elimination of All Forms of Discrimination against Women
CI: Confidence Interval
EDHS: Ethiopian Demographic and Health Survey
GBV: Gender-Based Violence
IPV: Intimate partner violence
OR: Odds Ratio
OI: Opportunistic infection
SPSS: Statistical package for social sciences
